# *mcr-1* in *Enterobacteriaceae* from Companion Animals, Beijing, China, 2012–2016

**DOI:** 10.3201/eid2304.161732

**Published:** 2017-04

**Authors:** Lei Lei, Yang Wang, Stefan Schwarz, Timothy R. Walsh, Yanran Ou, Yifan Wu, Mei Li, Zhangqi Shen

**Affiliations:** China Agricultural University, Beijing, China (L. Lei, Y. Wang, Y. Ou, Y. Wu, M. Li, Z. Shen);; Freie Universität Berlin, Berlin, Germany (S. Schwarz);; Cardiff University, Cardiff, Wales, UK (T.R. Walsh, M. Li);; Iowa State University, Ames, Iowa, USA (Z. Shen)

**Keywords:** colistin resistance, antimicrobial resistance, bacteria, epidemiology, Enterobacteriaceae, Escherichia coli, mcr-1, vector-borne infections, zoonoses, China

## Abstract

To investigate the prevalence of the recently emerging colistin resistance gene *mcr-1* in *Enterobacteriaceae* among companion animals, we examined 566 isolates collected from cats and dogs in Beijing, China, during 2012–2016. Of these isolates, 49 (8.7%) were *mcr-1*–positive.

Multidrug-resistant and extensively drug-resistant gram-negative bacteria are a major threat to public health worldwide (*1*,*2*). The recent rapid dissemination of carbapenem-resistant *Enterobacteriaceae* has worsened this situation and further narrowed treatment options for infections caused by these bacteria (*3*). Colistin is a last-resort drug for treating carbapenem-resistant *Enterobacteriaceae* infections (*4*). In 2016, we identified the mobile colistin resistance gene *mcr-1* (*1*). Soon after its description, *mcr-1* was observed in *Enterobacteriaceae* from humans and food-producing animals in >30 countries on 5 continents (*5*).

A 2016 article reported that a 50-year-old man who worked in a pet store tested positive for *mcr-1*–harboring *E. coli* (*6*). Investigation identified 6 multidrug-resistant *mcr-1*–producing *E. coli* isolates in samples from 4 dogs and 2 cats in the pet store, indicating that the pathogens can be transmitted between humans and companion animals. So far, the prevalence of *mcr-1*-containing *Enterobacteriaceae* in companion animals is largely unknown. In our study, we focused on estimating the prevalence of *mcr-1* in *Enterobacteriaceae* of companion animal origin in Beijing, China, during 2012–2016, and investigated the presence of the *mcr-1* gene in pet foods purchased there.

In Beijing, the total number of registered dogs and cats is ≈1.2 million. We collected samples from both healthy and sick dogs and cats in Veterinary Teaching Hospital of China Agricultural University.

A total of 566 nonduplicate *Enterobacteriaceae* strains were isolated from 1,439 nasal and rectal swab samples collected from 1,254 dogs and 185 cats during 2012–2016. We also isolated 25 *Enterobacteriaceae* from 32 nasal swab samples from the pet owners. Because the food chain is among the main routes for humans and companion animals to acquire foodborne pathogens, we collected a small sample of pet foods (dog food, n = 30; cat food, n = 5) containing chicken as the main ingredient in Beijing during June–August 2016.

The species of all *Enterobacteriaceae* were determined by 16S rDNA sequencing and matrix-assisted laser desorption/ionization time-of-flight mass spectrometry of specimens cultured on brain–heart infusion agar plates containing 2 μg/mL colistin. A total of 79/566 (14.0%) of the *Enterobacteriaceae* isolates from companion animals were resistant to colistin: 56 *E. coli*, 16 *Klebsiella pneumoniae,* 5 *Enterobacter cloacae*, 1 *Enterobacter aerogenes,* and 1 *Shigella* spp. PCR amplification of *mcr-1* indicated that 8.7% (49/566) of *Enterobacteriaceae* and 62.0% (49/79) of colistin-resistant isolates harbored the *mcr-1* gene, 47 *E. coli* and 2 *K. pneumoniae*. Only 1 *E. coli* isolate from a pet owner was colistin-resistant and *mcr-1*–positive. The proportions of *mcr-1*–containing *E. coli* per year ranged from 6.1% to 14.3% (Figure).

**Figure:**
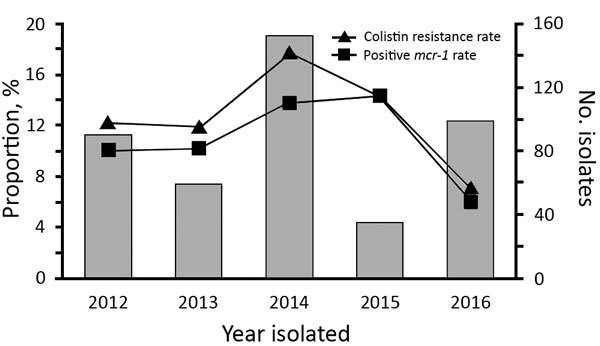
Proportion of colistin resistance and *mcr-1* in *Escherichia coli* of companion animal origin, Beijing, China, 2012–2016.

We examined the susceptibility of colistin-resistant *E. coli* to 8 other antimicrobial agents by agar dilution, according to the recommendations of Clinical and Laboratory Standards Institute (*7*). The *mcr-1*–carrying *E. coli* exhibited high resistance rates to ampicillin (97.9%), cefotaxime (91.5%), chloramphenicol (89.4%), and gentamicin (85.1%) (Technical Appendix Table 1) but were susceptible to imipenem. The *mcr-1*–positive *E. coli* were more often resistant to amoxicillin/clavulanate, ampicillin, and chloramphenicol than were the *mcr-1*–negative *E. coli* (p<0.05) (Technical Appendix Table 1).

All 57 colistin-resistant *E. coli* were subjected to XbaI pulsed-field gel electrophoresis (PFGE). The 55 colistin-resistant *E. coli* strains (2 nontypeable strains were excluded) were subdivided into 33 patterns and grouped into 31 clusters (A–Z, AB–AF) (online Technical Appendix Figure). The diversity and similarity of PFGE patterns of *E. coli* from different origins suggested that the dissemination of *mcr-1* was possibly related to both clonal expansion and horizontal transmission.

Of note, the 1 *E. coli* colistin-resistant, *mcr-1*–positive isolate from a pet owner had the same PFGE pattern as 5 isolates from dogs and cats. Multilocus sequence typing linked these 6 strains to sequence type 101. These results suggest that *E. coli* strains can be exchanged between companion animals and humans.

The PCR and sequence analysis of the pet food samples showed that 7 of 35 samples were positive for *mcr-1*. Companies in China produced 5 of these foods; the other 2 were from Italy and Belgium (Technical Appendix Table 2). These results suggest that pet foods may be a source from which intestinal bacteria of companion animals can acquire the *mcr-1* gene.

Currently, colistin is not used to treat companion animals in China. The companion animals included in this study were from an urban area of Beijing, so they had minimal or no contact with food-producing animals in which colistin may have been used. Because we found *mcr-1* in pet foods, we speculate that the pet food industry may be a source of *mcr-1* among companion animals. Because of frequent and close contact between humans and companion animals, our study proposes that opportunities exist to transmit colistin-resistant *Enterobacteriaceae* to and from both groups. Thus, colistin-resistant *Enterobacteriaceae* from companion animals may represent a potential risk to human health. Further surveillance and control efforts are needed to reduce colistin-resistant and *mcr-1*–containing *Enterobacteriaceae* in companion animals and food-producing animals.

Technical Appendix Antimicrobial drug MICs of colistin-resistant *Escherichia coli* from companion animals, presence of *mcr-1* gene in pet foods, and pulsed-field gel electrophoretic analysis of colistin-resistant *E. coli*.
