# Supplementary material for: mcr-1 in Enterobacteriaceae from Companion Animals, Beijing, China, 2012–2016
Source: Emerg Infect Dis. 2017 Apr;23(4):710–1. doi: 10.3201/eid2304.161732 (PMC5367392; doi:10.3201/eid2304.161732)
Supplement: Technical Appendix — Antimicrobial drug MICs of colistin-resistant Escherichia coli from companion animals, presence of mcr-1 gene in pet foods, and pulsed-field gel electrophoretic analysis of colistin-resistant E. coli. [file 16-1732-Techapp-s1.pdf]

Article DOI: http://dx.doi.org/10.3201/eid2304.161732

mcr-1 in *Enterobacteriaceae* from Companion Animals, Beijing, China, 2012–2016

Technical Appendix

Technical Appendix Table 1. Antimicrobial drug MICs of colistin-resistant *Escherichia coli* from companion animals (n=56)*

	mcr-1–positive *E. coli* (n = 47)			*mcr-1*–negative E. coli (n = 9)			
Antimicrobial agents†	MIC_{50}, µg/mL	MIC_{90}, µg/mL	Resistance, %	MIC_{50}, µg/mL	MIC_{90}, µg/mL	Resistance, %	p value‡
Colistin	8	256	100.0%	4	µg/mL	100.0%	NA
Polymyxin B	8	64	100.0%	4	16	100.0%	NA
Amoxicillin-clavulanic acid	64/32	>128/64	70.2%	16/8	16	33.3%	0.0296
Ampicillin	>256	>256	97.9%	128	64/32	66.7%	0.0111
Tigecycline	0.5	1	2.1%	0.25	>256	0.0%	NA
Enrofloxacin	32	64	74.5%	0.25	0.5	44.4%	0.1121
Cefotaxime	>256	>256	91.5%	128	128	66.7%	0.0740
Chloramphenicol	128	>256	89.4%	4	>256	22.2%	0.0002
Gentamicin	>256	>256	85.1%	64	>256	55.6%	0.0632
Imipenem	0.25	0.5	0.0%	0.25	>256	0.0%	NA

*Colistin-resistant *Escherichia coli* was isolated from 57 samples, including 1 from an owner of a companion animal that was excluded from this table; NA, not applicable.
†The MICs of amoxicillin-clavulanate, ampicillin, enrofloxacin, cefotaxime, chloramphenicol, gentamicin, and imipenum were interpreted according to the CLSI document VET01S 3rd ed. The MIC of colistin, polymixin B, and tigecycline were interpreted according to the European Committee on Antimicrobial Susceptibility Testing clinical breakpoints (version 6.0).
‡p values were determined by Fisher exact test.

Techncial Appendix Table 2. Characterization of pet foods and the presence of *mcr-1* gene

Type of pet food	Year of production	Ingredients	Pet food sample numbers*	Origin	*mcr-1*-positive pet food
Dry foods	2015	Chicken meal	15–19, 22	China	16, 19
Dry foods	2015	Chicken meal	20	Belgium	20
Dry foods	2015	Chicken meal	27	Australia	0
Dry foods	2015	Chicken, fish/pork	21, 23, 27, 29, 30, 34, 35	China	21, 34, 35
Dry foods	2016	Chicken	24–26	China	0
Dry foods	2015	Chicken	31, 32	USA	0
Dry foods	2015	Fish/chicken	33	Germany	0
Semi-moist foods	2016	Chicken	1,2,5	China	0
Semi-moist foods	2016	Chicken, bovine spleen	6–8	China	0
Semi-moist foods	2016	Chicken, pork/fish	3, 4	China	0
Canned wet foods	2016	Chicken, beef, mutton	9–11	Thailand	0
Canned wet foods	2016	Chicken	12–14	Italy	14

*Total: 35 samples of pet foods. Numbers 2, 4, 5, 33, and 34 are cat food; remaining samples are dog food

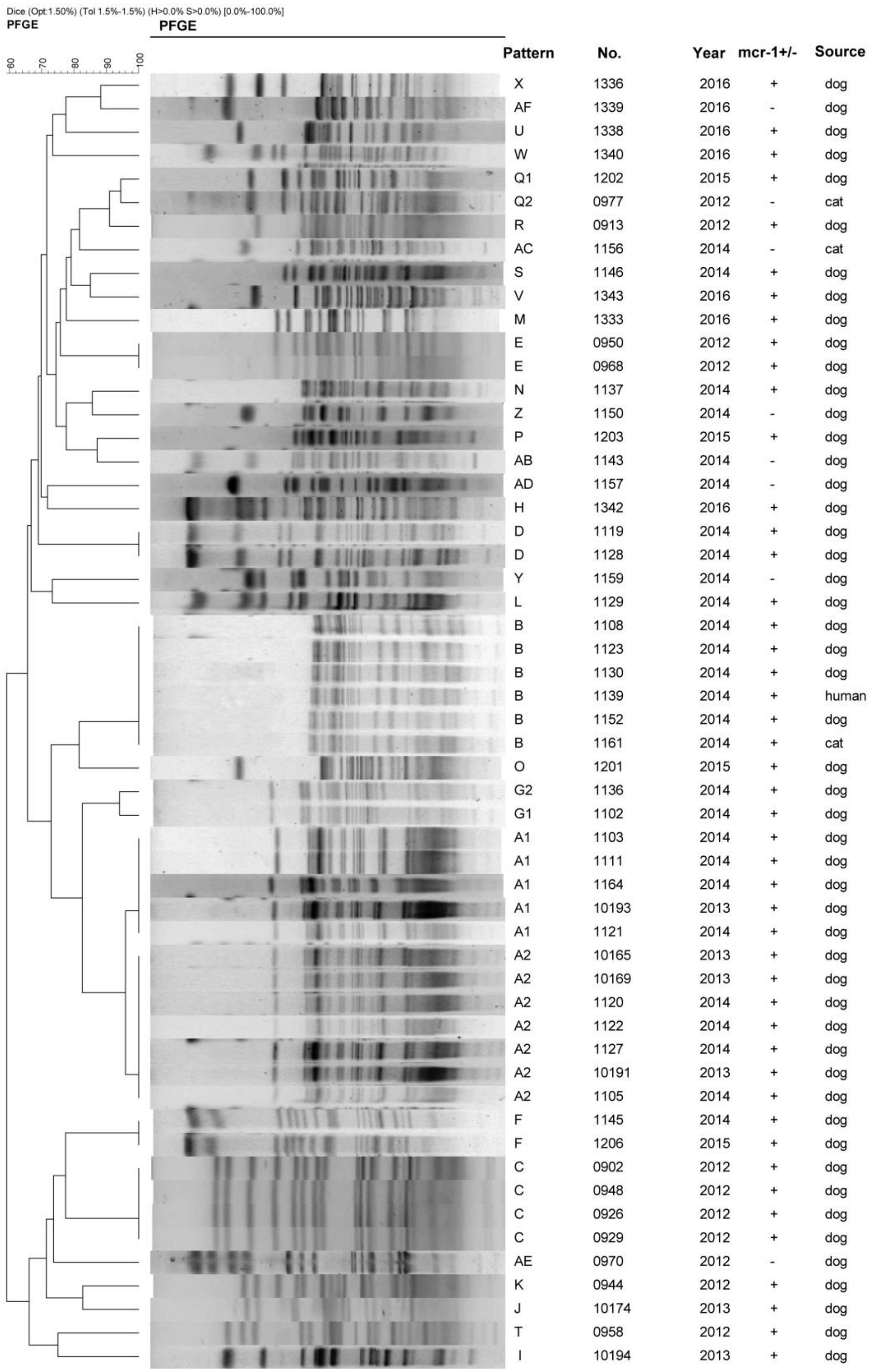

Pattern	No.	Year	mcr-1+/-	Source
X	1336	2016	+	dog
AF	1339	2016	-	dog
U	1338	2016	+	dog
W	1340	2016	+	dog
Q1	1202	2015	+	dog
Q2	0977	2012	-	cat
R	0913	2012	+	dog
AC	1156	2014	-	cat
S	1146	2014	+	dog
V	1343	2016	+	dog
M	1333	2016	+	dog
E	0950	2012	+	dog
E	0968	2012	+	dog
N	1137	2014	+	dog
Z	1150	2014	-	dog
P	1203	2015	+	dog
AB	1143	2014	-	dog
AD	1157	2014	-	dog
H	1342	2016	+	dog
D	1119	2014	+	dog
D	1128	2014	+	dog
Y	1159	2014	-	dog
L	1129	2014	+	dog
B	1108	2014	+	dog
B	1123	2014	+	dog
B	1130	2014	+	dog
B	1139	2014	+	human
B	1152	2014	+	dog
B	1161	2014	+	cat
O	1201	2015	+	dog
G2	1136	2014	+	dog
G1	1102	2014	+	dog
A1	1103	2014	+	dog
A1	1111	2014	+	dog
A1	1164	2014	+	dog
A1	10193	2013	+	dog
A1	1121	2014	+	dog
A2	10165	2013	+	dog
A2	10169	2013	+	dog
A2	1120	2014	+	dog
A2	1122	2014	+	dog
A2	1127	2014	+	dog
A2	10191	2013	+	dog
A2	1105	2014	+	dog
F	1145	2014	+	dog
F	1206	2015	+	dog
C	0902	2012	+	dog
C	0948	2012	+	dog
C	0926	2012	+	dog
C	0929	2012	+	dog
AE	0970	2012	-	dog
K	0944	2012	+	dog
J	10174	2013	+	dog
T	0958	2012	+	dog
I	10194	2013	+	dog

Technical Appendix Figure. XbaI pulsed-field gel electrophoretic analysis of colistin-resistant *Escherichia coli.*
